# Attachment, Emotion Dysregulation, and Physical IPV in Predominantly Hispanic, Young Adult Couples

**DOI:** 10.3390/ijerph18147241

**Published:** 2021-07-06

**Authors:** Deanna L. Pollard, Arthur L. Cantos

**Affiliations:** Department of Psychological Science, University of Texas Rio Grande Valley, Edinburg, TX 78539, USA; arthur.cantos@utrgv.edu

**Keywords:** attachment, impulsivity, emotion dysregulation, intimate partner violence (IPV), couples, relationships, Hispanic populations, Mexican American populations

## Abstract

Insecure attachment has been found to be a risk factor for perpetrating physical intimate partner violence (IPV). However, this association is likely exacerbated by additional factors, such as conflicting insecure attachment in one’s partner and difficulties with overall emotion regulation and impulse control. The present study aimed to examine the associations between insecure attachment and physical IPV perpetration in male and female partners, as well as to examine whether these associations are exacerbated by involvement with a partner with opposing attachment needs and overall emotion dysregulation and impulsivity. Additionally, this study examined whether partners’ emotion dysregulation interacted to predict IPV. Two hundred eight heterosexual couples primarily recruited from a Hispanic-serving university completed questionnaires on attachment, emotion dysregulation, and one’s own and one’s partner’s perpetration. Results revealed that attachment anxiety, impulsivity, and an interaction effect between attachment avoidance and partner’s attachment anxiety were associated with self-reported, but not partner-reported, male perpetration. For females, attachment anxiety was associated with female IPV (self-reported and partner-reported), and impulsivity was associated with self-reported female IPV. Overall, results underscore how relationships between known risk factors and IPV perpetration may differ depending on if IPV perpetration is measured using self-reported or partner-reported data. Additional results and implications are discussed.

## 1. Introduction

Physical intimate partner violence (IPV) consists of physical violence aimed at a current or former intimate partner [1]. It is a serious public health concern impacting millions of Americans each year and may result in severe injury or death [2]. Although IPV has been traditionally viewed as gender asymmetrical with male partners as the sole perpetrator and female partners as the sole victim [3,4], research has found women to report perpetrating IPV at equal or slightly higher rates than men, although IPV perpetrated by men is more likely to result in injury [5,6,7,8,9]. Further, bidirectional IPV (i.e., IPV where both partners are perpetrators and victims) has been found to be prevalent among college, community, and criminal justice samples [8]. Consequently, many researchers have adopted alternative theoretical models to the traditional gender-specific power and control model to better understand why physical IPV occurs [10]. One theoretical framework for IPV receiving increasing attention is Finkel’s I^3^ (pronounced “cubic”) theory, as it allows for the integration of several models to better understand how known risk factors interact to predict violence [10,11,12].

The three main factors in I^3^ theory are instigators, impelling factors, and inhibiting factors (i.e., the three I’s in I^3^ theory) [12]. Instigators are situational factors that may elicit an aggressive response from a person, such as disparaging remarks made by an intimate partner during conflict or experiencing rejection [13]. Impelling factors are dispositional or situational factors that increase one’s likelihood of experiencing an aggressive impulse in the presence of an instigating event, and inhibiting factors are those factors that override one’s urge to respond aggressively to instigating events [13]. According to Finkel [11,12], when violence-impelling factors are strong and violence-inhibiting factors are weak for at least one intimate partner during an instigating event, violence is likely to occur.

### 1.1. Violence-Impelling Factors: Attachment Anxiety and Attachment Avoidance 

Two plausible causal factors for perpetrating violence that—from a I^3^ perspective—can be viewed as violent-impelling factors are the two insecure attachment dimensions, attachment anxiety and attachment avoidance [10,13,14,15,16,17,18]. To better understand these factors, a brief overview of attachment theory and how it may relate to violence is provided below.

#### 1.1.1. Attachment Theory

Bowlby [19,20,21] asserted that all humans are born with a biopsychological system called the attachment system, which is designed to aid in proximity seeking with an attachment figure (i.e., significant others) during times of physiological or emotional distress [22]. According to attachment theory, people who had consistently and adequately responsive attachment figures throughout their life develop positive internal working models of themselves and others [22]. These individuals perceive themselves as worthy of care and support and perceive others as dependable. However, people whose early attachment figures were inconsistently responsive or irresponsive to one’s needs for care and support develop negative internal working models of themselves, others, or both. Bowlby primarily focused on the attachment bond between mother and child; however, several researchers have applied attachment theory to better understand adult romantic attachment bonds [23,24]. Adult romantic attachment theory asserts that there are two dimensions of insecure attachment, attachment anxiety and attachment avoidance [22,23]. Attachment anxiety reflects the extent to which individuals ruminate over their adult attachment figures abandoning or rejecting them, whereas attachment avoidance reflects the extent to which individuals are uncomfortable with emotional intimacy [23]. Individuals who have a lot of attachment anxiety, attachment avoidance, or both in their adult relationships are considered to have an insecure attachment to their adult attachment figures, whereas those who have little to no attachment anxiety or attachment avoidance are considered to have a secure attachment style. Securely attached individuals are both comfortable with independence and relying on their partners during times of need. These individuals acknowledge even their most unpleasant emotions and address their relationship concerns with their partners in constructive ways to resolve issues and foster emotional intimacy in their relationship [22].

Instead of approaching conflict constructively, individuals characterized by insecure attachment utilize defensive secondary strategies to alleviate uncomfortable emotions regarding their intimate relationships [22]. Individuals with a lot of attachment anxiety utilize a defensive secondary strategy referred to as hyperactivation of the attachment system to achieve their desired level of closeness to an attachment figure who is perceived as insufficiently physically or emotionally available. This strategy consists of intensifying negative emotions, becoming hypersensitive to cues of rejection and abandonment, jealousy, and engaging in clingy and coercive behaviors [22]. Individuals with a lot of attachment avoidance utilize a defensive secondary strategy referred to as deactivation of the attachment system to achieve their desired level of distance from an attachment figure who is perceived as overwhelmingly close. Deactivation of the attachment system consists of suppressing and refusal to acknowledge uncomfortable emotions, disregarding inclinations to reach out to one’s partner, and determination to tackle one’s issues alone [22]. Individuals with a lot of attachment anxiety and attachment avoidance are thought to utilize some combination of hyperactivating and deactivating strategies to alleviate emotional discomfort in their adult relationships [23].

From an attachment perspective, interpersonal anger arises from frustrated attachment needs, which results in the frustrated individual engaging in behaviors that could be conceptualized as protest behaviors directed at a partner who is perceived as not satisfying the frustrated individual’s needs for proximity or space [25]. If the frustrated partner expresses their anger constructively, then the anger may be functional to the relationship as it alerts their attachment figure of the relationship threat perceived by the frustrated partner. If the attachment figure is aware of the issue, they may attempt to resolve the issue to meet their partner’s needs better. However, interpersonal anger becomes problematic if it manifests in dysfunctional ways, such as emotional or physical abuse. Thus, Bowlby [26] viewed interpersonal violence as an exaggerated form of something that—if manifested in a nonviolent way—could actually be functional toward the maintenance of attachment bonds.

#### 1.1.2. The Interaction of Partners’ Insecure Attachment Needs and Violence

Pistole [27] postulated that frustrated attachment needs might be an ongoing relational issue for partners with different attachment styles. For example, if one partner has a lot of attachment anxiety and the other partner has a lot of attachment avoidance, the anxiously attached partner’s way of responding to perceived relational threats may inadvertently trigger the avoidantly attached partner’s attachment system and vice versa, resulting in the escalation of conflict. Pistole’s theory is in line with qualitative data from heterosexual partners examining the association between insecure attachment and IPV revealing that pursue–distance struggles often preceded violence [28]. Indeed, Finkel [11] stated that dyadic processes, such as the demand–withdraw pattern—can influence the extent to which partners experience violence-impelling and -inhibiting forces during conflict, as research has shown such processes can elevate chances of violence [29]. Therefore, involvement in a relationship with a partner with opposing insecure attachment needs may create a relationship dynamic that serves as a risk factor for experiencing weak violence-inhibiting forces for individuals who have an insecure attachment style because of their increased susceptibility of experiencing escalated conflict regarding pursue–distance struggles.

#### 1.1.3. Research on Attachment and IPV in Couples

Research has found an association between insecure attachment and IPV [6,30,31,32,33], with most of the few studies including attachment and IPV data from both partners revealing an avoidant–anxious partner attachment pattern—particularly, attachment avoidance in men and attachment anxiety in women—to be a predictor of IPV for men and women [34,35,36,37]. For example, Bond and Bond [35] found the combination of anxious attachment in women and dismissing (i.e., high avoidance and low anxiety) attachment in men to predict a prevalence of overall IPV victimization. Similarly, in a sample of 70 community couples, Doumas et al. [36] found interactions between high attachment avoidance in men and high attachment anxiety in women to predict male-perpetrated and female-perpetrated IPV.

Although research provides support for opposing insecure attachment in both partners influencing the prevalence of male and female IPV perpetration in couples, the current literature has several limitations worth mentioning. First, Bond and Bond [35] and Doumas et al. [36] both recruited small samples (*N* = 41 couples and *N* = 70 couples, respectively). Second, even though Doumas et al. [36] were interested in how attachment dimensions related to IPV, they used a categorical attachment questionnaire (i.e., a questionnaire that assigns participants to a specific attachment group) and converted participants’ scores into the two insecure attachment dimensions instead of using a more reliable measure that assesses the two attachment dimensions directly, such as the ECR or its revised version [38,39]. Third, to mitigate the influence of underreporting violence, Roberts and Noller [37] and Doumas et al. [36] combined self-reports and partner reports of male and female IPV to create their male and female perpetration variables. For example, Roberts and Noller treated any report of male violence either from the male or female partner as an indication that male IPV was present and vice versa, whereas Doumas and colleagues treated the higher report for each couple as indicative of their most accurate response on a given IPV item. For instance, if the male partner in a couple indicated that he engaged in a specific IPV act “two to three times” in the past year, but his female partner reported that he engaged in that act “more than once a month” in the past year, Doumas et al. used “more than once a month” as the male partner’s response for that item. Although altering partners’ perpetration and victimization reports as a way to lessen the influence of underreporting violence is a common practice in studies including data from both partners [40], and underreporting of perpetration does occur [41,42], several IPV concordance studies have found intimate partners to also disagree on the occurrence of male and female IPV in their relationships because men [43,44] or women [45,46,47] overreported their own perpetration in comparison to their partners’ victimization reports. Thus, it may be preferable to include two male and female IPV measures, one based on self-reports and the other on partner reports (four outcome variables total), when working with dyadic data [40]. However, no study examining the associations between insecure attachment and physical IPV in couples has assessed male and female IPV this way. Lastly, most research on attachment and IPV conducted in the United States has been conducted on predominantly White samples. Since the U.S. Hispanic population has been increasing [48], it is important for researchers to determine whether findings regarding known risk factors, such as insecure attachment, and IPV replicate to Hispanic couples to better address the needs of these couples. Furthermore, terms such as “Hispanic” and “Latinx” are used to describe individuals from various cultural backgrounds with little commonalities except for their language [49]. Thus, research should not only attempt to determine whether findings regarding known risk factors and IPV replicate on Hispanic samples, but also differentiate between U.S. Hispanic subpopulations when doing so.

One of the aims of the present study is to examine the interaction effect of attachment avoidance in male partners and anxious attachment in female partners on physical IPV in couples by addressing the limitations mentioned. Specifically, we (1) recruited a larger sample of couples, (2) used a more appropriate attachment questionnaire that allowed us to measure attachment anxiety and attachment avoidance directly, (3) included self-report and partner-report measures of male and female physical IPV, and (4) recruited a predominantly Hispanic (Mexican American) sample.

### 1.2. Weak Violence-Inhibiting Factors: Emotion Dysregulation and Impulsivity

Overall emotion dysregulation encompasses difficulties regulating impulses; difficulties engaging in goal-directed behavior when experiencing negative emotions; low perceptions of one’s ability to effectively engage in emotion regulation strategies; and lack of clarity, acceptance, and awareness of one’s emotions [50]. Overall emotion dysregulation and impulsivity have been found to relate to IPV perpetration and moderate relationships between IPV and other known risk factors, such as trait anger and negative affect [13,50,51,52].

Aside from involvement with an insecurely attached partner with opposing attachment needs, insecurely attached individuals who also exhibit difficulties with impulse control and/or overall emotion dysregulation may be at elevated risk for acting on aggressive impulses in the presence of an instigating event [11,13]. However, little research has examined how impulse control or overall emotion regulation abilities interact with insecure attachment to predict physical IPV. One study conducted on a female U.S. college student sample found that, in line with I^3^ theory, anxious attachment was only associated with physical IPV perpetration among college student women who also reported high rates of overall emotion dysregulation [53]. However, Bell et al. [53] did not examine whether emotion dysregulation interacted with avoidant attachment, nor did they examine these associations on college student men. Thus, another aim of this study is to examine whether emotion dysregulation and impulsivity interact with attachment anxiety and attachment avoidance to predict male and female IPV perpetration.

In addition to strengthening the associations between insecure attachment dimensions and IPV perpetration, the association between emotion dysregulation and IPV perpetration may also be impacted by one’s partner’s emotion regulation abilities. For example, in a sample of 160 adult couples, Lee et al. [54] found men’s own emotion dysregulation to relate to men’s self-reported physical IPV perpetration if their intimate partner also had difficulties regulating emotions. Thus, an additional aim of the present study was to also examine interactions between partners’ emotion regulation difficulties and self-reported and partner-reported male and female physical perpetration.

### 1.3. The Current Study

The purpose of the present study is to examine the relationships between attachment, emotion dysregulation, impulsivity, and male and female physical IPV perpetration in a sample primarily comprising Hispanic young adult couples. Specifically, this study aims to (1) examine the relationships between attachment dimensions and male- and female-perpetrated physical IPV, (2) examine whether there are interaction effects between men’s attachment avoidance and women’s attachment anxiety on male- and female-perpetrated physical IPV, (3) examine the relationships between overall emotion dysregulation and impulsivity on male- and female-perpetrated physical IPV, (4) examine whether there are interaction effects between attachment dimensions and emotion regulation variables (overall emotion dysregulation and impulsivity) on male- and female-perpetrated physical IPV, and (5) examine whether there are interaction effects between partners’ emotion regulation variables and physical IPV perpetration. This study contributes to the limited research examining known IPV risk factors on understudied U.S. subpopulations by examining whether findings from the previously mentioned research replicate in a sample primarily comprising Hispanic couples while simultaneously addressing some of the limitations of the current literature. The hypotheses are as follows.

#### 1.3.1. Hypothesis 1

Because previous research has found attachment anxiety and attachment avoidance to predict IPV perpetration for both men and women, it is hypothesized that attachment anxiety and attachment avoidance would significantly predict male-perpetrated and female-perpetrated physical IPV.

#### 1.3.2. Hypothesis 2

Second, because previous research has found interaction effects between high attachment avoidance in men and high attachment anxiety in women to predict male-perpetrated and female-perpetrated physical IPV, it is hypothesized that there will be interaction effects between men’s attachment avoidance scores and women’s anxious attachment scores on male and female IPV perpetration.

#### 1.3.3. Hypothesis 3

Consistent with previous literature, it is hypothesized that difficulties regulating emotions (overall emotion dysregulation and impulsivity) would predict physical IPV perpetration in men and women.

#### 1.3.4. Hypothesis 4

In line with Finkel’s I^3^ theory, it is hypothesized that difficulties with emotion regulation (i.e., overall emotion dysregulation and impulsivity) will moderate the associations between insecure attachment and physical IPV perpetration in men and women, with high emotion regulation difficulties exacerbating the association between insecure attachment dimensions and physical IPV perpetration.

#### 1.3.5. Hypothesis 5

In line with Lee et al. [54], it is hypothesized that there would be interaction effects between partners’ emotion dysregulation variables and male- and female-perpetrated physical IPV.

Due to the lack of research examining the associations among insecure attachment, emotion dysregulation, and physical IPV perpetration in U.S. Hispanic samples (let alone dyadic U.S. Hispanic samples), it is unclear whether results obtained in the present sample would differ from those obtained in samples drawn from other U.S. populations.

## 2. Materials and Methods

All study materials and procedures were approved by The University of Texas Rio Grande Valley’s Institutional Review Board (IRB).

### 2.1. Participants

Two hundred forty-three couples were recruited through (1) an undergraduate psychology research participation pool at a large Hispanic-serving university located in a predominantly Mexican American region of the U.S., (2) flyers posted throughout the university, or (3) flyers posted on Facebook. Eligible participants had to be at least 18 years of age, involved in a monogamous (i.e., not an open relationship) romantic relationship, and both partners must have been willing to participate. Of the 243 couples recruited, 2 couples were excluded for reporting involvement in an open relationship and 18 were removed because partners disagreed on relationship demographics such as relationship status, relationship length, and whether they are cohabiting. Same-sex couples were excluded due to there only being 15 of them. Therefore, only 208 heterosexual couples (416 individuals in total) were included in the analyses. Of the 208 couples included in the analyses, 85.1% (*n* = 177) of couples consisted of two partners who identified as Hispanic, 12% (*n* = 25) of couples consisted of one partner who identified as Hispanic, and 2.9% (*n* = 6) of couples were not Hispanic. The mean age for men was 21.68 (SD = 4.04), and the mean age for women was 20.74 (SD = 3.51). The majority of men (72.1%, *n* = 150) and women (86.5%, *n* = 180) were college students. The majority of couples were involved in a committed dating relationship (89.4%, *n* = 186) and did not live with each other (78.8%, *n* = 164). Of the couples, 43.8% had been together for more than 2 years (*n* = 91), 22.1% had been together for 1 to 2 years (*n* = 46), 13.9% (*n* = 29) had been together for 6 months to 1 year, 18.3% had been together for 1 to 6 months (*n* = 38), and 1.9% (*n* = 4) had been together for less than 1 month.

### 2.2. Measures

*Demographics.* Participants completed a demographic survey that consisted of questions inquiring on participants’ age, sexual orientation, ethnicity, employment status, college student status, college student classification, socioeconomic status, relationship status, relationship length, and whether they currently lived with their partner.

*Attachment Dimensions.* We used The Experiences in Close Relationships Revised Questionnaire (ECR-R) [39] to assess romantic attachment. The ECR-R is the revised version of The Experiences in Close Relationships Questionnaire (ECR) [38], a continuous adult romantic attachment assessment. The ECR-R consists of 36 items that ask the participant to indicate, on a 7-point Likert scale (1 = strongly disagree to 7 = strongly agree), the degree to which they believe each item reflects how they experience their intimate relationships. The ECR-R has two 18-item subscales, one that assesses attachment anxiety and another that assesses attachment avoidance. Higher scores on the Attachment Anxiety subscale indicate greater attachment anxiety in intimate relationships, whereas higher scores on the Attachment Avoidance subscale indicate higher attachment avoidance in intimate relationships. Both ECR-R subscales demonstrated strong test–retest reliability over 6 weeks [55] and strong convergent and discriminant validity [56]. Internal consistencies reported for the Attachment Anxiety and Attachment Avoidance ECR-R subscales are typically over 0.90 [57]. In the present study, internal consistencies for the Attachment Anxiety and Attachment Avoidance subscales were 0.90 and 0.87 for women and 0.90 and 0.90 for men.

*Emotion Dysregulation and Impulsivity.* The Difficulties in Emotion Regulation Scale (DERS) [50] composite score was used to measure overall emotion dysregulation and the DERS Impulse subscale was used to assess impulsivity. The DERS consists of 36 items that assess different aspects of emotion regulation difficulties, namely nonacceptance of emotions, difficulties engaging in goal-directed behavior, lack of emotional clarity, difficulties with impulse control, perceived lack of access to emotion regulation strategies, and lack of awareness of one’s emotions. The participant is asked to indicate on a 5-point Likert scale (1 “almost never” to 5 “almost always”) the extent to which each item best applies to them. Higher scores on the overall assessment indicate greater overall difficulties regulating emotions, and higher scores on the Impulsivity subscale (items 37, 31, 17, 23, 4, and 28) indicate greater difficulty with impulse control. The overall DERS and the Impulse subscale both demonstrated good internal consistency with Cronbach’s alpha coefficients of 0.93 and 0.86, respectively, and the overall DERS demonstrated good test–retest reliability over a 4–8-week period [50]. In the present study, the internal consistencies for the overall DERS and impulse subscale scores were 0.94 and 0.90 for women and 0.93 and 0.87 for men.

*Intimate Partner Violence.* IPV was assessed using participants’ prevalence scores on the Physical Assault Scale of the Conflict Tactics Scale Revised (CTS2) [58]. The CTS2 is the revised version of the original CTS [59]. The Physical Assault Scale of the CTS2 is a 12-item, 7-point Likert-type scale (0 “This has never happened” to 6 “More than 20 times in the past year”) that assesses the frequency of perpetration of various acts of minor and severe forms of physical IPV by participants and their partners. The CTS2 Physical Assault Scale demonstrated good internal consistency with a Cronbach’s alpha coefficient of 0.86 [58]. In the present study, the internal consistency coefficients for the CTS2 Physical Assault Scale were 0.78 for women and 0.80 for men.

Since the current study did not recruit a clinical sample and most couples are likely to report no incidences of violence, CTS2 Physical Assault Scale scores were dichotomized. Following Straus et al.‘s [58] instructions, participants’ CTS2 physical assault perpetration prevalence scores were calculated by dichotomizing participants’ continuous CTS2 physical assault perpetration scores (0 = no perpetration, 1 = perpetrated IPV at least once). Likewise, participants’ prevalence CTS2 physical assault victimization scores were created by dichotomizing participants’ continuous victimization scores (0 = no victimization, 1 = victim of at least one act of IPV). Men’s perpetration prevalence scores and women’s victimization prevalence scores were used as our measures of self-reported and partner-reported male-perpetrated physical IPV, and women’s perpetration prevalence scores and men’s victimization prevalence scores were used as our measures of self-reported and partner-reported female-perpetrated physical IPV.

### 2.3. Procedures

#### 2.3.1. Couples who Participated Before COVID-19

Couples who participated before the coronavirus 2019 (COVID-19) pandemic met with the first author or a research assistant in a reserved computer lab on campus, and all questionnaires were compiled into one online survey. First, the person proctoring the session went over the consent form with each partner and provided them with an ID. This ID consisted of the initials of the research personnel proctoring the session, followed by a number and the letter A or B. Each partner in each couple received the same number but men’s IDs ended with a B and women’s IDs ended with an A (e.g., one couple was DP1A and DP1B, whereas another couple was DP2A and DP2B). The purpose of these IDs was so that we could match partners’ data together without needing participants’ names. After consent was obtained and IDs assigned, the proctor emailed the study survey link to the partner who was the primary contact (in most couples, this partner was the one participating for course credit) and instructed that individual to send the survey link to their partner. After both partners confirmed that they received the survey link, they were instructed to sit on opposite ends of the computer lab and enter their assigned ID into the text box that appeared at the beginning of their surveys. After that, participants proceeded to complete the ECR-R, the DERS, the CTS2, and the demographic questions.

#### 2.3.2. Couples who Participated during COVID-19

Couples who participated during the COVID-19 pandemic participated exclusively online. At least one partner of each couple who participated during the pandemic accessed the survey link through the study’s profile page on the undergraduate participation pool website or a Facebook post that included a flyer of the study and the survey link. After consent was obtained via an online consent form included in the survey link, participants were asked whether they were currently involved in an intimate relationship and whether they were aware of the study’s requirement that their partner must also be willing to participate in the study. After responding to those questions, the study’s survey link appeared on the screen with a note instructing the participant to take a moment to send the survey link to their partner if they had not done so already. Next, participants were asked to enter in the first letter of their first name, the first letter of their last name, their date of birth, the first letter of their partner’s first name, the first letter of their partner’s last name, and their partner’s date of birth. The purpose of these items is so that we could match partners’ data together without including participants’ full names. After that, participants completed the ECR-R, the DERS, the CTS2, and the demographic questions.

To examine whether differences in pre-COVID and during-COVID procedures had an effect on couples’ IPV reports, a dichotomous variable named COVID was created (0 = participated before COVID and 1 = participated during COVID). Chi-square results revealed there were no significant differences in self-reported male IPV (χ^2^(1, *N* = 208) = 0.07, *p* = 0.94), partner-reported male IPV (χ^2^(1, *N* = 208) = 0.11, *p* = 0.75), self-reported female IPV (χ^2^(1, *N* = 208) = 1.34, *p* = 0.25), and partner-reported female IPV (χ^2^(1, *N* = 208) = 0.07, *p* = 0.79) among couples who participated before or during the COVID-19 pandemic. Thus, differences in pre-COVID and during-COVID study procedures appeared to not have affected how couples responded to CTS2 items.

### 2.4. Data Analytic Plan

The IBM Statistical Package for the Social Sciences (SPSS) version 27 (International Business Machines Corporations, Armonk, NY, USA) was used to conduct all analyses. As Kenny et al. [60] recommended, for every couple, male and female partners’ data were entered in a single SPSS case so that the dyad—instead of each participant—can be treated as the unit of analysis.

#### 2.4.1. Preliminary Analyses

First, descriptive statistics were conducted to examine means, standard deviations, and frequency percentages for demographic and study variables. Second, bivariate correlations were conducted to examine correlations among IPV perpetration, study variables, and continuous demographics (i.e., relationship length and men and women’s age), and chi-square tests of independence were conducted to examine associations among all IPV variables. Lastly, chi-square tests of independence were conducted to determine if differences in IPV exist between cohabiting and non-cohabiting couples. All demographic variables found to be associated with IPV variables were included as covariates in primary analyses.

#### 2.4.2. Primary Analyses

A series of hierarchical logistic regressions were conducted to address this study’s hypotheses. Because overall emotion dysregulation was measured using DERS composite scores and impulsivity was measured using the impulse subscale of the DERS, overall emotion dysregulation and impulsivity were never included in the same regression models. Significant interactions were probed using Hayes’ Macro PROCESS v3.5 SPSS extension for regression analyses (developed by Dr. Andrew Hayes [61], the software can be downloaded from https://www.processmacro.org/index.html) to determine the nature of the interactions. To better compare results obtained in the present study with previous studies examining interactions between partners’ attachment dimensions on IPV, hypothesis 2 for male and female perpetration was also examined using combined reports of male and female perpetration (i.e., 1 = either partner reported male/female IPV, 0 = no partner reported male/female IPV).

## 3. Preliminary Results

Means, standard deviations, and percentages for demographic and study variables are displayed in Table 1.

### 3.1. Bivariate Correlations

Bivariate correlation results (Table 2, Table 3, Table 4, Table 5, Table 6 and Table 7) revealed that all men and women’s attachment variables were positively correlated with one another (*p* < 0.01).

#### 3.1.1. Male Perpetration

Self-reported male physical IPV was positively correlated with attachment anxiety (r = 0.20, *p* < 0.01), attachment avoidance (r = 0.17, *p* < 0.05), overall emotion dysregulation (r = 0.15, *p* < 0.05), and impulsivity (r = 0.17, *p* < 0.05) in men and attachment anxiety (r = 0.23, *p* < 0.01) in women. In contrast, partner-reported male physical IPV was not correlated with men’s attachment dimensions, overall emotion dysregulation, or impulsivity. Instead, partner-reported male physical IPV was positively correlated with attachment anxiety (r = 0.15, *p* < 0.05), overall emotion dysregulation (r = 0.15, *p* < 0.05), and impulsivity (r = 0.21, *p* < 0.01) in women. Thus, associations between men’s insecure attachment dimensions, emotion dysregulation variables, and male perpetration were only further examined in binary logistic regression analyses using self-reported male perpetration as the outcome variable. Neither relationship length nor age (male or female) was correlated with self-reported or partner-reported male perpetration (*p* > 0.05).

#### 3.1.2. Female Perpetration

Both self-reported and partner-reported female physical IPV were positively correlated with attachment anxiety in women (r = 0.23, *p* < 0.01 and r = 0.18, *p* < 0.01, respectively) but uncorrelated with women’s attachment avoidance and men’s characteristics (*p* > 0.05). Therefore, associations between women’s attachment avoidance and female perpetration were not further examined with binary logistic regression analyses. Additionally, self-reported, but not partner-reported, female physical IPV was positively correlated with women’s overall emotion dysregulation (r = 0.28, *p* < 0.01) and impulsivity (r = 0.29, *p* < 0.01) scores. Thus, associations between women’s emotion dysregulation/impulse control and female perpetration were only further examined in binary logistic regression analyses using self-reported female IPV as the outcome variable. Similar to male perpetration, neither relationship length nor age was correlated with self-reported or partner-reported female perpetration.

### 3.2. Associations among IPV Variables and Prevalence of IPV

Chi-square tests of independence revealed that all male-perpetrated and female-perpetrated IPV variables were significantly associated with one another (*p* ≤ 0.001; Table 6).

Among the 208 men and 208 women in our sample, 20.2% (*n* = 42) of men and 29.8% (*n* = 62) of women reported perpetrating at least one act of physical IPV in the past year (Table 1). Among the 42 men who reported perpetrating at least one act of physical IPV, 76.2% (*n* = 32) also reported being victims of past-year IPV, despite that only 50% (*n* = 21) of their female partners reported perpetrating. Among the 62 women who reported perpetrating past-year IPV, 69.4% (*n* = 43) also reported being victims, despite that only 33.9% (*n* = 21) of their male partners reported perpetrating IPV. Thus, it is apparent that there is a great deal of disagreement between partners regarding the occurrence of male-perpetrated and female-perpetrated physical IPV in the past year.

### 3.3. Comparing Cohabiting and Non-cohabiting Couples on IPV

Overall, greater proportions of men and women in cohabiting versus non-cohabiting relationships reported an occurrence of male-perpetrated and female-perpetrated physical IPV (Table 7). However, chi-square results revealed that there were only significant differences in the proportions of men who reported perpetrating and being victimized by past-year physical IPV between cohabiting and non-cohabiting couples (χ^2^(1, *N* = 208) = 4.68, *p* < 0.05 and χ^2^(1, *N* = 208) = 6.49, *p* < 0.05, respectively). Thus, involvement in a cohabiting relationship was only included as a covariate in analyses where self-reported male IPV and partner-reported female IPV were the outcome variables.

## 4. Primary Results

### 4.1. Male Perpetration

#### 4.1.1. Hypothesis 1

The first hypothesis for male perpetration was that men’s attachment anxiety and attachment avoidance scores on the ECR-R would be associated with male IPV. Because the bivariate correlation between men’s attachment anxiety and men’s attachment avoidance scores was high (r = 0.56, *p* < 0.01), it is likely that there would be shared variance between them that could explain male-perpetrated IPV. Thus, separate regressions were conducted to examine the associations between male IPV and the two attachment dimensions. Results for self-reported male IPV revealed that, while controlling for involvement in a cohabiting relationship, both attachment anxiety (*B* = 0.44, *p* < 0.01) and attachment avoidance (*B* = 0.45, *p* < 0.05) in men predicted self-reported male perpetration. The model including attachment anxiety accounted for 10% (Nagelkerke *R*^2^ = 0.10) of the variance in self-reported male perpetration, and the model including attachment avoidance accounted for 8% (Nagelkerke *R*^2^ = 0.08) of the variance in self-reported male perpetration.

#### 4.1.2. Hypothesis 2

The second hypothesis for male perpetration was that women’s attachment anxiety scores would interact with men’s attachment avoidance scores to predict male IPV.

**Figure 1:**
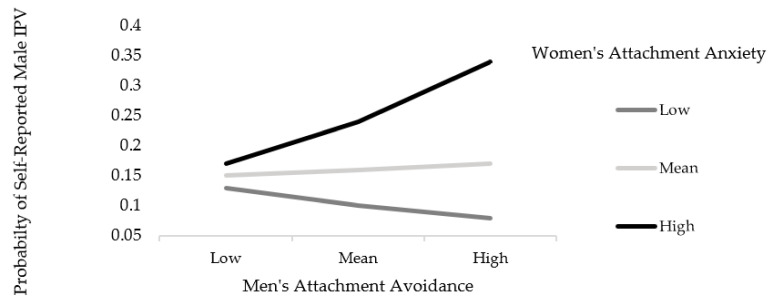
Interaction effect between men’s attachment avoidance and women’s attachment anxiety on self-reported male IPV.

*Self-Reported Male IPV.* Results for self-reported male IPV revealed that, while controlling for involvement in a cohabiting relationship, there was a significant interaction effect between men’s attachment avoidance and women’s attachment anxiety (*B* = 0.33, *p* < 0.05), indicating that women’s attachment anxiety moderated the relationship between men’s attachment avoidance and self-reported male perpetration. The simple slopes analysis revealed that higher scores on attachment avoidance in men were only significantly associated with self-reported male perpetration if their female partners scored above the mean in attachment anxiety (*B* = 0.49, *p* < 0.05; see Figure 1). The model that included the interaction term accounted for 16% (Nagelkerke *R*^2^ = 0.16) of the variance in self-reported male perpetration.

*Partner-Reported Male IPV.* Results for partner-reported male IPV revealed that women’s attachment anxiety alone was significantly associated with partner-reported male IPV (*B* = 0.30, *p* < 0.05). However, women’s attachment anxiety only accounted for 3% (Nagelkerke *R*^2^ = 0.03) of the variance in partner-reported male IPV.

*Combined-Reported Male IPV.* Results revealed that when male IPV was measured using combined reports of male IPV, there was no significant interaction between men’s attachment avoidance and women’s attachment anxiety on male physical perpetration (*B* = 0.17, *SE* = 0.48, *p* = 0.24).

#### 4.1.3. Hypothesis 3

The third hypothesis for male perpetration was that men’s overall emotion dysregulation scores and men’s impulsivity scores on the DERS would be associated with male perpetration. Results revealed that, while controlling for involvement in a cohabiting relationship, men’s overall emotion dysregulation and men’s impulsivity were both associated with self-reported male perpetration (*B* = 0.02, *p* = 0.02 and *B* = 0.08, *p* = 0.02, respectively), with the model including overall emotion dysregulation and the model including impulsivity accounting for 7% (Nagelkerke *R*^2^ = 0.7) and 8% (Nagelkerke *R*^2^ = 0.8) of the variance in self-reported male IPV, respectively.

#### 4.1.4. Hypothesis 4

The fourth hypothesis for male perpetration was that men’s overall emotion dysregulation and impulsivity scores would interact with men’s attachment anxiety and attachment avoidance to predict male IPV. Results for self-reported male IPV revealed that while controlling for involvement in a cohabiting relationship, the association between men’s attachment anxiety and self-reported male perpetration was not moderated by men’s overall emotion dysregulation (*B* = 0.00, *p* = 0.54) or men’s impulsivity (*B* = 0.00, *p* = 0.91). Similarly, the association between men’s attachment avoidance and self-reported male perpetration was also not moderated by men’s overall emotion dysregulation (*B* = 0.00, *p* = 0.95) and men’s impulsivity (*B* = 0.03, *p* = 0.47).

#### 4.1.5. Hypothesis 5

The fifth hypothesis for male perpetration was that women’s overall emotion dysregulation and impulsivity scores would interact with men’s emotion regulation variables to predict male IPV. Results for self-reported male IPV revealed that there was not a significant interaction between women’s overall emotion dysregulation and men’s overall emotion dysregulation on self-reported male perpetration (*B* = 0.00, *p* = 0.32). Similarly, there was no significant interaction between women’s impulsivity and men’s impulsivity on self-reported male IPV (*B* = 0.00, *p* = 0.98).

#### 4.1.6. Final Models for Male-Perpetrated IPV

The final models for self-reported and partner-reported male perpetration are displayed in Table 8.

*Self-Reported Male IPV.* Although men’s overall emotion dysregulation and impulsivity were associated with self-reported male IPV beyond the effect of involvement in a cohabiting relationship (see Section 4.1.3), they were not associated with self-reported male IPV beyond the effect of men’s anxious attachment (*B* = 0.01, *p* < 0.36 and *B* = 0.05, *p* < 0.13, respectively). Similarly, overall emotion dysregulation was not associated with self-reported male IPV beyond the effect of men’s avoidant attachment (*B* = 0.01, *p* < 0.15). However, men’s impulsivity remained significantly associated with self-reported male IPV beyond the effect of male attachment avoidance (*B* = 0.07, *p* < 0.05). Thus, a binary logistic regression was conducted with men’s impulsivity, men’s attachment avoidance, women’s attachment anxiety, and the men’s attachment avoidance X women’s attachment anxiety interaction term as the predictor variables (Table 8). Results revealed that men’s impulsivity (*B* = 0.07, *p* < 0.05) and the men’s attachment avoidance X women’s attachment anxiety interaction term (*B* = 0.34, *p* < 0.05) were significantly associated with self-reported male IPV. This model explained 19% of the variance in self-reported male perpetration.

*Partner-Reported Male IPV.* Only women’s attachment anxiety predicted partner-reported male IPV (*B* = 0.30, *p* < 0.05).

### 4.2. Female Perpetration

#### 4.2.1. Hypothesis 1

The first hypothesis for female perpetration was that women’s attachment anxiety and attachment avoidance scores on the ECR-R would be associated with female perpetration.

*Self-Reported Female IPV.* Results revealed that women’s attachment anxiety was significantly associated with self-reported female IPV (*B* = 0.42, *p* < 0.01) and explained 7% (Nagelkerke *R*^2^ = 0.07) of the variance in self-reported female perpetration. 

*Partner-Reported Female IPV.* Similarly, results for partner-reported female IPV revealed that, after controlling for involvement in a cohabiting relationship, women’s attachment anxiety was significantly associated with partner-reported female IPV (*B* = 0.40, *p* < 0.01). The overall model explained 10% (Nagelkerke *R*^2^ = 0.10) of the variance.

#### 4.2.2. Hypothesis 2

The second hypothesis for female perpetration was that men’s attachment avoidance scores would interact with women’s attachment anxiety scores to predict female perpetration.

*Self-Reported Female IPV.* Results revealed that there was no significant interaction effect between women’s attachment anxiety and men’s attachment avoidance on self-reported (*B* = −0.18, *p* = 0.83) female perpetration.

*Partner-Reported Female IPV.* Similarly, there was also no significant interaction effect between women’s attachment anxiety and men’s attachment avoidance on partner-reported female perpetration before (*B* = 0.28, *p* = 0.06) and after (*B* = 0.22, *p* = 0.15) controlling for involvement in a cohabiting relationship.

*Combined-Reported Female IPV.* When female IPV was measured using partners’ combined reports, results revealed that there was no significant interaction between women’s attachment anxiety and men’s attachment avoidance on female physical perpetration (*B* = 0.15, *p* = 0.29).

#### 4.2.3. Hypothesis 3

The third hypothesis for female perpetration was that women’s overall emotion dysregulation scores and women’s impulsivity scores on the DERS would be associated with female perpetration. Results revealed that women’s overall emotion dysregulation (*B* = 0.02, *p* < 0.001) and impulsivity (*B* = 0.10, *p* < 0.001) were significantly associated with self-reported female-perpetrated IPV. Both regressions explained 11% (Nagelkerke *R*^2^ = 0.11) of the variance in self-reported female IPV.

#### 4.2.4. Hypothesis 4

The fourth hypothesis for female perpetration was that women’s overall emotion dysregulation and impulsivity scores would interact with women’s attachment anxiety and attachment avoidance scores to predict female perpetration. Results revealed that neither women’s overall emotion dysregulation (*B* = 0.00, *p* = 0.36) nor women’s impulsivity (*B* = −0.04, *p* = 0.10) moderated the association between women’s attachment anxiety and self-reported female IPV.

#### 4.2.5. Hypothesis 5

The fifth hypothesis for female perpetration was that men’s overall emotion dysregulation and impulsivity scores would interact with women’s emotion regulation variables to predict female IPV. Results revealed that there was no significant interaction effect between women’s overall emotion dysregulation and men’s overall emotion dysregulation on self-reported female perpetration (*B* = 0.00, *p* = 0.66). Similarly, there was no significant interaction between women’s impulsivity and men’s impulsivity on self-reported female IPV (*B* = 0.00, *p* = 0.86).

#### 4.2.6. Final Models Predicting Female IPV

The final models predicting self-reported and partner-reported female IPV are displayed in Table 9.

*Self-Reported Female IPV.* Although women’s impulsivity did not interact with women’s anxious attachment to predict self-reported female IPV (see Section 4.2.5), women’s attachment anxiety and women’s impulsivity were independently associated with self-reported female IPV (*B* = 0.33, *p* < 0.05 and *B* = 0.09, *p* < 0.001, respectively; Table 9) and explained 12% of the variance (Nagelkerke *R*^2^ = 0.12). In contrast, not only was the women’s emotion dysregulation X women’s attachment anxiety interaction term an insignificant predictor of self-reported female IPV (see Section 4.2.5.), but women’s attachment anxiety was no longer associated with self-reported female IPV when overall women’s emotion dysregulation was included in the model (*B* = 0.24, *p* = 0.11; Table 9). The model including women’s attachment anxiety and women’s overall emotion dysregulation as the predictor variables explained 15% of the variance (Nagelkerke *R*^2^ = 0.15).

*Partner-Reported Female IPV.* When female perpetration was measured using partner reports, only women’s attachment anxiety—and not women’s overall emotion dysregulation or impulsivity—was associated with perpetrating physical IPV, *B* = 0.40, *p* < 0.01.

## 5. Discussion

As predicted, both attachment and emotional regulation difficulties predict intimate partner violence perpetration in both males and females. However, the observed relationships are different for male and female perpetration and are also dependent on whether the relationships between these variables and IPV perpetration are based on self or partner reports.

Both attachment avoidance and attachment anxiety predicted self-reported male IPV perpetration irrespective of whether the partners were living together or not. However, in males, attachment avoidance was only predictive of perpetration in those cases where the female partner was high in anxious attachment. Both overall emotional dysregulation and more specifically impulsivity were predictive of self-reported male IPV perpetration. Neither overall emotional dysregulation nor impulsivity affected the relationship between attachment and self-reported IPV perpetration in males. However, a model including cohabitation, impulsivity, male attachment avoidance, and the interaction between female attachment anxiety and male attachment avoidance predicted 19% of the variance in male self-perpetration. When male perpetration was assessed by partner reports, only female attachment anxiety was found to predict male perpetration.

Only female attachment anxiety was found to predict both self- and partner-reported female IPV, and this relationship was not dependent on male attachment characteristics. Overall emotional dysregulation and impulsivity were both predictive of female self-reported IPV perpetration, and a model including both attachment anxiety and impulsivity accounted for 15% of the variance.

In summary, both males and females who are anxiously attached are at greater risk of perpetrating violence against their partner and, in addition, males who are avoidantly attached have a greater risk of perpetrating violence against their partner if their female partner is anxiously attached. Both emotional dysregulation and impulsivity also increase the risk of perpetrating violence against an intimate partner in both males and females. When either partner has difficulties with both attachment and impulsivity, the risk is increased. Only anxious attachment in females was predictive of male or female partner-reported IPV.

The findings from this study are largely consistent with findings from previous literature which found avoidantly attached men to be more likely to perpetrate IPV if they were romantically involved with an anxiously attached partner [35,36]. However, when male perpetration was assessed using partner reports, only anxious attachment in women—and not insecure attachment in men nor the interaction between men’s avoidant attachment and women’s anxious attachment—was associated with male perpetration. This finding highlights the importance of assessing both self and partner reports of male perpetration given that the obtained relationships are dependent on which member of the dyad is reporting.

With respect to emotional regulation, the results obtained when assessing male perpetration using self-reports are consistent with previous research suggesting that overall emotion dysregulation and impulsivity predict IPV perpetration [49,51], but the results obtained when assessing male perpetration with partner reports were not. Inconsistent with I^3^ theory which asserts that a weak violence-inhibiting factor such as emotion dysregulation/impulsivity would moderate the association between a violence-impelling factor (e.g., attachment anxiety/avoidance) and IPV perpetration, neither overall emotional dysregulation nor impulsivity moderated the relationship between men’s attachment scores and self-reported male IPV perpetration. However, a model including cohabitation, impulsivity, female attachment anxiety, and the interaction between female attachment anxiety and male attachment avoidance predicted 19% of the variance in male self-reported perpetration. Thus, it may be that difficulties with impulse control and insecure attachment are both associated with, but do not have an interacting effect on, male perpetration. Furthermore, inconsistent with Lee et al. [54], women’s emotion regulation abilities did not moderate the associations between men’s emotion regulation abilities and male perpetration. This difference is interesting given the methodological similarities between the present study and the study of Lee et al. [54]. Both Lee et al. [54] and the present study assessed emotion dysregulation using the DERS and recruited a sample primarily comprising undergraduate students and their dating partners. Additionally, Lee et al. reported a similar prevalence of self-reported male-perpetrated and female-perpetrated physical IPV to the prevalence reported in the present study (25% and 20.2% for male IPV and 33.8% and 29.8% for female IPV, respectively). However, there were two notable differences between this study and Lee et al.’s study which might account for the different results obtained. Firstly, this study used a regression approach to study the interaction between partner’s impulse control difficulties, and Lee et al. conducted actor–partner interdependence models (APIMs). Secondly, a notable difference between the two is that most couples included in Lee et al.’s analyses were White (92.5%), whereas most couples included in the present study’s analyses were Hispanic (85.1%). It could be that cultural differences exist in how partners’ emotion regulation abilities impact relationship conflict management. However, future research examining interactions between partners’ emotion dysregulation on IPV in Hispanic couples is needed to understand better whether this difference can be attributed to differences in ethnicity.

Results for female perpetration revealed that only female attachment anxiety predicted both self- and partner-reported female IPV, and male attachment characteristics did not moderate these relationships. This finding is consistent with research demonstrating a relationship between anxious attachment and IPV perpetration in women [32] but inconsistent with research suggesting that involvement with an avoidantly attached partner moderates the association between anxious attachment and female perpetration [35,36,37]. It is unclear why the interaction between attachment avoidance in men and attachment anxiety in women did not predict female IPV in the present study. Unexpectedly, the present study also found that men’s attachment avoidance did not moderate the association between women’s anxious attachment and female perpetration when female IPV was assessed using a combined report of female perpetration. This finding suggests that the present study’s use of self- and partner-report measures of female IPV does not explain why the men’s avoidant attachment X women’s anxious attachment interaction effect found in previous research was not replicated in the present study. However, discrepancies in results between the present study and previous research could be due to other methodological differences. First, previous studies examining attachment and IPV in couples were conducted on predominantly White samples [35,36,37], whereas the sample included in the present study was over 80% Hispanic. Second, the sample in the present study primarily consisted of undergraduate students and their partners in their early 20s, whereas other studies primarily recruited older, clinical [35], and community [36,37] couples. Third, most of the research suggesting that the attachment avoidance–attachment anxiety couple attachment combination predicts female IPV were conducted on samples smaller than the one included in the present study [35,36], so results of previous studies may not be stable enough to be replicated in larger samples. Furthermore, the attachment measure used in the current study differed from those utilized in previous studies. This study is the first study to examine the relationships between partners’ attachment dimensions and physical IPV by using the ECR-R to assess insecure attachment continuously. Future studies examining interactions between partners’ attachment dimensions on large samples of college student couples and Hispanic community or clinical couples where attachment dimensions are assessed using the ECR or its revised version are needed to interpret these differences in findings better.

Concerning the associations between emotion dysregulation, attachment, and female perpetration, overall emotional dysregulation and impulsivity were both predictive of female self-reported IPV perpetration, and a model including both attachment anxiety and impulsivity accounted for 15% of the variance. However, similar to results for males, overall emotion dysregulation and impulsivity did not moderate the association between anxious attachment and self-reported female perpetration, nor did they interact with men’s overall emotion dysregulation and impulsivity scores to predict female perpetration.

Regarding relationships between demographic variables and IPV in the present study, consistent with literature demonstrating cohabiting couples are at greater risk for experiencing IPV, partner-reported female perpetration and self-reported male perpetration were correlated with involvement in a cohabiting relationship [62]. However, male-perpetrated and female-perpetrated IPV (self-reported and partner-reported) were not correlated with relationship length or age in the present sample. This is inconsistent with literature demonstrating younger age and longer relationship duration to be associated with greater IPV [36,47,62]. With respect to age, it could be that there was not enough variability in age in the present sample, nor were there enough couples reporting a prevalence of IPV perpetration/victimization, for a correlation between age and IPV to occur since most participants were between the ages of 18 and 21. With respect to relationship length, it is important to note that 78.8% of couples in the current sample reported that they were not cohabiting. It is possible that relationship duration has less of an impact on IPV among couples who have yet to move in together, such as most of the couples included in this study. Length of relationship may not matter as much as whether the couple is living together or not, which suggests the actual amount of time spent together and commitment to the relationship may have more of an impact on IPV perpetration rather than how long they have been together.

### 5.1. Clinical Implications

Both men and women who have developed difficulties with attachment—very likely due to exposure to adversity in childhood, not having had their emotional needs met consistently and reliably, and exposure to interparental violence—are at risk of perpetrating violence in their adult intimate relationships. In addition, both men and women who develop emotional regulation difficulties, especially impulsivity, are also at risk of perpetrating violence in their adult intimate relationships. The abilities to have attachment needs met and regulate one’s emotions are essential skills necessary for successful communication in intimate relationships. The present study’s results suggest that individuals who lack these skills are ill-poised to have successful and satisfying relationships. These deficits are present in young adults and suggest that it would be necessary for prevention programs at the high school level, or even as early as elementary school, to address both attachment issues and emotional regulation skills in order to reduce the probability that these individuals would go on to perpetrate violence in their intimate relationships. It would also be necessary for treatment programs for individuals who have already perpetrated violence towards their intimate partner to address both attachment issues and the ability to regulate emotions. Discussions regarding childhood relationships with parents and their impact on perpetrators’ intimate relationships would be beneficial to include in treatment. Moreover, teaching emotional regulation skills, whether done via cognitive behavioral therapy, the recent Achieving Change Through Value-Based Therapy program (ACTV) [63], or a combination of both treatment modalities, is likely to be beneficial in both primary prevention and education programs for perpetrators. It is also imperative to recognize that not all perpetrators have deficits in attachment and/or emotional regulation [6] and that in order to maximize the potential to help perpetrators of IPV and reduce recidivism, it would be important to teach these skills to individuals who evidence such deficits to tailor the treatment programs to the individual needs of the perpetrators.

### 5.2. Strengths

An important strength of this study is its focus on couples. Both attachment and violence perpetration are assessed with both members of the dyad. This allowed the researchers to study whether the different ways that partners met their attachment needs increased the risk of violence perpetration. Findings revealed that, indeed, for males who tried to meet their attachment needs by withdrawing emotionally, if their partner was anxiously attached and as a result resorted to seeking increased proximity when their attachment needs were threatened, they were more likely to report perpetrating IPV. Addressing this issue in treatment programs designed to reduce recidivism would likely result in a reduction in recidivism as it would directly target the pursue–distance struggles that may lead to escalated violent conflict. This finding also highlights the importance of including not only perpetrators but also the partners during pretreatment assessment, as well as suggesting that couples experiencing IPV may benefit from both members of the dyad participating in treatment in those cases where mismatched insecure attachment styles are present.

Additionally, perpetration and victimization of violence were assessed in both members of the same dyad. Results also showed that concordance rates in self-reports of violence perpetration were very low, with men being more likely to underreport both their own and their partner’s violence perpetration. It is impossible to determine which report is more accurate. The importance of this finding lies in highlighting the need to assess both male and female reports given that the obtained relationships with other variables depend on which report is used.

### 5.3. Limitations

Although the present study added to the current literature on attachment, emotion dysregulation, and IPV in heterosexual couples, it is not without its limitations. The present study was conducted on a sample primarily recruited from a large Hispanic-serving institution in the Rio Grande Valley (RGV), a region in the U.S. state of Texas that is over 90% Hispanic and of Mexican American origin [64]. This is both a strength and limitation of the study. It is a strength in that it is the first study to examine the relationships between heterosexual partners’ attachment dimensions and IPV in Hispanic couples, but a limitation in that the findings of this study cannot be generalized to clinical or community Hispanic couples in the RGV or other regions of the U.S. Additionally, because the current study was mostly conducted on college students and their partners and thus most couples did not report violence, participants’ CTS2 physical perpetration and victimization scores were dichotomized. Although dichotomized IPV variables provide meaningful information regarding the prevalence of and risk factors for perpetrating IPV at least once, dichotomized scores leave out other meaningful information such as how frequently one engages in violence and what factors are associated with more frequent or more severe violence. Additional limitations of the current research are its cross-sectional design and use of questionnaires (both self-report and partner-report questionnaire data are subject to bias); moreover, although partners who participated before the COVID-19 pandemic were seated away from one another in a computer lab, there is no way of knowing whether partners who participated during the pandemic were in close proximity while completing their surveys. However, since chi-square results revealed no significant differences in IPV reports between couples who participated before or during the pandemic, this may not have had much of an impact on the way participants responded to the survey items. Future studies should address these limitations by replicating this research on predominantly Hispanic clinical or relationally distressed community couples and examine male and female IPV dichotomously and continuously. Previous research screening community couples for moderate to high relationship distress have found most couples to report IPV occurrences [65]. Thus, assessing male and female IPV as continuous variables may be appropriate in future research examining attachment and IPV in Hispanic community couples if couples are screened using similar methods [65]. Additionally, future research should expand on the current study by examining the relationships between partners’ attachment dimensions, emotion dysregulation, and other forms of IPV, such as sexual coercion and psychological abuse, by measuring them using self-reports and partner reports of male-perpetrated and female-perpetrated IPV.

## 6. Conclusions

Despite this study’s limitations, the present study adds to the current literature on heterosexual partners’ attachment dimensions, emotion dysregulation, and male-perpetrated and female-perpetrated IPV in several ways. First, this study examined these associations in a predominantly Hispanic sample. Second, this study measured physical IPV via self and partner reports. Third, attachment anxiety and attachment avoidance were assessed using the ECR-R, which measures these dimensions directly. Fourth, we recruited a larger sample than those recruited in some previous studies examining interactions between partners’ attachment dimensions on IPV. Hypotheses regarding associations between attachment dimensions, emotion dysregulation variables, and IPV were mostly but not totally supported. This study most notably underscored how the way perpetration is measured can impact the results obtained. Given self-report questionnaires, such as the CTS and its revised version, are the most commonly used tools for measuring IPV, researchers must be able to obtain reliable information regarding IPV when assessing IPV with these questionnaires. The results from the present study demonstrated that predictors of male-perpetrated and female-perpetrated IPV can differ substantially depending on whether IPV is measured via self or partner reports, which is problematic given most IPV research is based on reports from only one partner. Thus, future research should explore how the lack of interrater reliability between partners’ IPV reports can impact findings obtained regarding IPV risk factors.

## Figures and Tables

**Table 1:** Descriptive statistics for study and demographic variables.

Variables	*M* or *N* (SD or %)
Attachment Anxiety ^a^	
Men	2.73 (1.15) *
Women	2.98 (1.17) *
Attachment Avoidance ^a^	
Men	2.33 (0.96)
Women	2.17 (0.85)
Emotion Dysregulation	
Men	79.89 (23.68) ***
Women	88.84 (26.22) ***
Impulsivity	
Men	10.55 (4.87) ***
Women	13.14 (6.43) ***
Male Perpetration ^b^	
According to Men	42 (20.2%)
According to Women	52 (25%)
Female Perpetration ^b^	
According to Men	54 (26%)
According to Women	62 (29.8%)
Age ^a^	
Men	21.68 (4.04)
Women	20.74 (3.51)
College Students ^b^	
Men	150 (72.1%)
Women	180 (86.5%)
Hispanic Couples ^b^	
Both partners Hispanic	177 (85.1%)
One partner Hispanic	25 (12%)
Both partners are not Hispanic	6 (2.9%)
Cohabiting Couples ^b^	
Yes	44 (21.2%)
No	164 (78.8%)
Relationship status ^b^	
Dating Relationship	186 (89.4%)
Engaged	6 (2.9%)
Married	16 (7.7%)
Relationship length ^b^	
More than 2 years	91 (43.8%)
Between 12 and 24 months	46 (22.1%)
Between 6 and 12 months	29 (13.9%)
Between 1 and 6 months	38 (18.3%)
Less than 1 month	4 (1.9%)

*Note:*^a^—means and standard deviations are displayed. ^b^—sample sizes and percentages are displayed. * *p* < 0.05. *** *p* < 0.001.

**Table 2:** Bivariate correlations among self-reported male intimate partner violence (IPV), relationship length, age, attachment, emotion dysregulation, and impulsivity.

Variables	1	2	3	4	5	6	7	8	9	10	11	12
1. SR Male IPV												
2. Relationship Length	0.11											
3. Male Age	0.05	0.13										
4. Female Age	−0.01	0.20 **	0.78 **									
5. Male Attach Anxiety	0.20 **	−0.01	0.06	0.00								
6. Male Attach Avoid	0.17 *	−0.13	0.11	0.07	0.56 **							
7. Female Attach Anxiety	0.23 **	−0.07	−0.15 *	−0.17 *	0.39 **	0.36 **						
8. Female Attach Avoid	0.05	−0.15 *	−0.03	−0.05	0.25 **	0.29 **	0.47 **					
9. Male Emo. Dysreg	0.15 *	−0.02	−0.04	−0.10	0.53 **	0.42 **	0.23 **	0.19 **				
10. Female Emo. Dysreg	0.06	−0.03	−0.08	−0.14 *	0.21 **	0.14 *	0.47 **	0.31 **	0.18 **			
11. Male Impulsivity	0.17 *	0.07	0.01	−0.01	0.33 **	0.12	0.14 *	0.12	0.70 **	0.19 **		
12. Female Impulsivity	0.04	0.02	−0.01	−0.06	0.11	0.13	0.24 **	0.18 *	0.14 *	0.70 **	0.16 *	

* *p* < 0.05. ** *p* < 0.01. SR = self-reported.

**Table 3:** Bivariate correlations among partner-reported male IPV, relationship length, age, attachment, emotion dysregulation, and impulsivity.

Variables	1	2	3	4	5	6	7	8	9	10	11	12
1. PR Male IPV												
2. Relationship Length	0.08											
3. Male Age	−0.01	0.13										
4. Female Age	−0.04	0.20 **	0.78 **									
5. Male Attach Anxiety	0.10	−0.01	0.06	0.00								
6. Male Attach Avoid	0.11	−0.13	0.11	0.07	0.56 **							
7. Female Attach Anxiety	0.15 *	−0.07	−0.15 *	−0.17 *	0.39 **	0.36 **						
8. Female Attach Avoid	0.08	−0.15 *	−0.03	−0.05	0.25 **	0.29 **	0.47 **					
9. Male Emo. Dysreg.	0.10	−0.02	−0.04	−0.10	0.53 **	0.42 **	0.23 **	0.19 **				
10. Female Emo. Dysreg.	0.15 *	−0.03	−0.08	−0.14 *	0.21 **	0.14 *	0.47 **	0.31 **	0.18 **			
11. Male Impulsivity	0.08	0.07	0.01	−0.01	0.33 **	0.12	0.14 *	0.12	0.70 **	0.19 **		
12. Female Impulsivity	0.21 **	0.02	−0.01	−0.06	0.11	0.13	0.24 **	0.18 *	0.14 *	0.70 **	0.16 *	

* *p* < 0.05. ** *p* < 0.01. PR = partner-reported.

**Table 4:** Bivariate correlations among self-reported female physical IPV, relationship length, age, attachment, emotion dysregulation, and impulsivity.

Variables	1	2	3	4	5	6	7	8	9	10	11	12
1. SR Female Physical IPV												
2. Relationship Length	0.12											
3. Female Age	−0.02	0.20 **										
4. Male Age	−0.01	0.13	0.78 **									
5. Female Attachment Anxiety	0.23 **	−0.07	−0.17 *	−0.146 *								
6. Female Attachment Avoidance	0.10	−0.15 *	−0.05	−0.03	0.47 **							
7. Male Attachment Anxiety	0.13	−0.01	0.00	0.06	0.39 **	0.25 **						
8. Male Attachment Avoidance	0.10	−0.13	0.07	0.11	0.36 **	0.29 **	0.56 **					
9. Female Emo. Dysreg.	0.28 **	−0.03	−0.14 *	−0.08	0.47 **	0.31 **	0.21 **	0.14 *				
10. Male Emo. Dysreg.	0.10	−0.02	−0.10	−0.04	0.23 **	0.19 **	0.53 **	0.42 **	0.18 **			
11. Female Impulsivity	0.29 **	0.02	−0.06	−0.01	0.24 **	0.18 *	0.11	0.13	0.70 **	0.14 *		
12. Male Impulsivity	0.11	0.07	−0.01	0.01	0.14 *	0.12	0.33 **	0.12	0.19 **	0.70 **	0.16 *	

* *p* < 0.05. ** *p* <0.01. SR = Self-Reported.

**Table 5:** Bivariate correlations among partner-reported female IPV, relationship length, age, attachment, emotion dysregulation, and impulsivity.

Variables	1	2	3	4	5	6	7	8	9	10	11	12
1. PR Female Physical IPV												
2. Relationship Length	0.12											
3. Female Age	0.03	0.20 **										
4. Male Age	0.09	0.13	0.78 **									
5. Female Attachment Anxiety	0.18 **	−0.07	−0.17 *	−0.15 *								
6. Female Attachment Avoid	−0.01	−0.15 *	−0.05	−0.03	0.47 **							
7. Male Attachment Anxiety	0.10	−0.01	0.00	0.06	0.39 **	0.25 **						
8. Male Attachment Avoid	0.09	−0.13	0.07	0.11	0.36 **	0.29 **	0.56 **					
9. Female Emo. Dysreg.	0.12	−0.03	−0.14 *	−0.08	0.47 **	0.31 **	0.21 **	0.14 *				
10. Male Emo. Dysreg.	0.08	−0.02	−0.10	−0.04	0.23 **	0.19 **	0.53 **	0.42 **	0.18 **			
11. Female Impulsivity	0.11	0.02	−0.06	−0.01	0.24 **	0.18 *	0.11	0.13	0.70 **	0.14 *		
12. Male Impulsivity	0.10	0.07	−0.01	0.01	0.14 *	0.12	0.33 **	0.12	0.19 **	0.70 **	0.16 *	

* *p* < 0.05. ** *p* < 0.01. PR = partner-reported.

**Table 6:** Chi-square tests of independence results for IPV variables.

Variables	*χ* ^2^
SR Male IPV and SR Female IPV	10.26 ***
PR Male IPV and PR Female IPV	17.64 ***
SR Female IPV and PR Male IPV	92.68 ***
SR Male IPV and PR Female IPV	69.10 ***
SR Male IPV and PR Male IPV	33.45 ***
SR Female IPV and PR Female IPV	14.21 ***

SR = self-reported. PR = partner-reported. *** *p* ≤ 0.001.

**Table 7:** Proportions of self-reported and partner-reported male and female IPV perpetration across cohabiting and non-cohabiting couples.

	Cohabiting	Not Cohabiting	*χ* ^2^
IPV Variables	% (*n*)	% (*n*)	
SR Male IPV	31.8 (14)	17.1 (28)	4.68 *
PR Male IPV	34.1 (15)	22.6 (37)	2.46
SR Female IPV	40.9 (18)	26.8 (44)	3.29
PR Female IPV	40.9 (18)	22.0 (36)	6.49 *

*N* = 208 couples. * *p* < 0.05. SR = self-reported. PR= partner-reported. CR = combined-reported.

**Table 8:** Binary logistic regression models: predictors of self-reported and partner-reported male physical IPV.

Variables	Nagelkerke *R*^2^	*B* (*SE*)	OR	95% CI for OR
SR Male IPV				
	0.10			
Cohabiting Relationship		0.96 (0.40)	2.60 *	(1.18, 5.71)
Male Attachment Anxiety		0.44 (0.15)	1.56 **	(1.17, 2.08)
	0.19			
Cohabiting Relationship		0.89 (0.43)	2.44 *	(1.05, 5.66)
Male Impulsivity		0.07 (0.04)	1.07 *	(1.00, 1.15)
Male Attachment Avoidance		0.07 (0.22)	1.08	(0.69, 1.67)
Female Attachment Anxiety		0.42 (0.18)	1.53 *	(1.08, 2.16)
Male Avoid X Female Anx.		0.34 (0.17)	1.41 *	(1.01, 1.97)
PR Male IPV				
	0.03			
Female Attachment Anxiety		0.30 (0.14)	1.35 *	(1.03, 1.76)

Lines in between indicate separate regression models. * *p* < 0.05. ** *p* < 0.01. SE = standard error. OR = odds ratio. CI = confidence interval. SR = self-reported. PR = partner-reported. Dependent variables are in bold.

**Table 9:** Binary logistic regression models: predictors of self-reported and partner-reported female physical IPV.

Variables	Nagelkerke *R*^2^	*B* (*SE*)	OR	95% CI for OR
SR Female IPV				
	0.07			
Female Attachment Anxiety		0.42 (0.13)	1.53 **	(1.17, 1.99)
	0.12			
Female Attachment Anxiety		0.24 (0.15)	1.27	(0.95, 1.71)
Female Emo. Dysreg.		0.02 (0.01)	1.02 ***	(1.01, 1.03)
	0.15			
Female Attachment Anxiety		0.33 (0.14)	1.40 *	(1.06, 1.84)
Female Impulsivity		0.09 (0.02)	1.09 ***	(1.04, 1.14)
PR Female IPV				
	0.10			
Cohabiting Relationship		1.06 (0.38)	2.88 **	(1.38, 6.02)
Female Attachment Anxiety		0.40 (0.14)	1.49 **	(1.14, 1.97)

* *p* < 0.05. ** *p* < 0.01. *** *p* < 0.001. SE = standard rrror. OR = odds ratio. CI = confidence interval. SR = self-reported. PR = partner-reported. Lines in between indicate separate regression models. Outcome variables are in bold.

## Data Availability

The data presented in this study are available on request from the first author.
